# Comparison of microarray expression profiles between follicular variant of papillary thyroid carcinomas and follicular adenomas of the thyroid

**DOI:** 10.1186/1471-2164-16-S1-S7

**Published:** 2015-01-15

**Authors:** Hans-Juergen Schulten, Zuhoor Al-Mansouri, Ibtisam Baghallab, Nadia Bagatian, Ohoud Subhi, Sajjad Karim, Hosam Al-Aradati, Abdulmonem Al-Mutawa, Adel Johary, Abdulrahman A Meccawy, Khalid Al-Ghamdi, Osman Abdel Al-Hamour, Mohammad Hussain Al-Qahtani, Jaudah Al-Maghrabi

**Affiliations:** 1 Center of Excellence in Genomic Medicine Research, King Abdulaziz University, Jeddah, Saudi Arabia; 2 KACST Technology Innovation Center in Personalized Medicine, King Abdulaziz University, Jeddah, Saudi Arabia; 3 Department of Pathology and Laboratory Medicine, King Faisal Specialist Hospital and Research Center, Jeddah, Saudi Arabia; 4 Department of Surgery, Faculty of Medicine, King Abdulaziz University, Jeddah, Saudi Arabia; 5 Department of Surgery, King Faisal Specialist Hospital and Research Center, Jeddah, Saudi Arabia; 6 Department of Pathology, Faculty of Medicine, King Abdulaziz University, Jeddah, Saudi Arabia

**Keywords:** whole-transcript oligonucleotide microarrays; expression profiling, follicular variant of papillary thyroid carcinoma (FVPTC), follicular adenoma (FA) of the thyroid.

## Abstract

**Background:**

Follicular variant of papillary thyroid carcinoma (FVPTC) and follicular adenoma (FA) are histologically closely related tumors and differential diagnosis remains challenging. RNA expression profiling is an established method to unravel molecular mechanisms underlying the histopathology of diseases.

**Methods:**

*BRAF* mutational status was established by direct sequencing the hotspot region of exon 15 in six FVPTCs and seven FAs. Whole-transcript arrays were employed to generate expression profiles in six FVPTCs, seven FAs and seven normal thyroid tissue samples. The threshold of significance for differential expression on the gene and exon level was a *p*-value with a false discovery rate (FDR) < 0.05 and a fold change cutoff > 2. Two dimensional average linkage hierarchical clustering was generated using differentially expressed genes. Network, pathway, and alternative splicing utilities were employed to interpret significance of expression data on the gene and exon level.

**Results:**

Expression profiling in FVPTCs and FAs, all of which were negative for a *BRAF* mutation, revealed 55 transcripts that were significantly differentially expressed, 40 of which were upregulated and 15 downregulated in FVPTCs *vs*. FAs. Amongst the most significantly upregulated genes in FVPTCs were *GABA B receptor*, *2* (*GABBR2*), *neuronal cell adhesion molecule* (*NRCAM*), *extracellular matrix protein 1* (*ECM1*), *heparan sulfate 6-O-sulfotransferase 2* (*HS6ST2*), and *retinoid X receptor*, *gamma* (*RXRG*). The most significantly downregulated genes in FVPTCs included *interaction protein for cytohesin exchange factors 1* (*IPCEF1*), *G protein-coupled receptor 155* (*GPR155*), *Purkinje cell protein 4* (*PCP4*), *chondroitin sulfate N-acetylgalactosaminyltransferase 1* (*CSGALNACT1*), and *glutamate receptor interacting protein 1* (*GRIP1*). Alternative splicing analysis detected 87 genes, 52 of which were also included in the list of 55 differentially expressed genes. Network analysis demonstrated multiple interactions for a number of differentially expressed molecules including vitamin D (1,25- dihydroxyvitamin D3) receptor (VDR), SMAD family member 9 (SMAD9), v-kit Hardy-Zuckerman 4 feline sarcoma viral oncogene homolog (KIT), and RXRG.

**Conclusions:**

This is one of the first studies using whole-transcript expression arrays to compare expression profiles between FVPTCs and FAs. A set of differentially expressed genes has been identified that contains valuable candidate genes to differentiate both histopathologically related tumor types on the molecular level.

## Introduction

Thyroid cancer is one of the most common cancer types in the Gulf region and is considered the second most common cancer in young Saudi women [1]. The majority of papillary thyroid carcinomas (PTCs) which represent approximately 80 % of all differentiated thyroid carcinomas are histopathologically classified as conventional PTCs. The follicular variant of PTC (FVPTC) represents the largest subtype and accounts for about 30 % of all PTCs [2]. Minor and rare subtypes include tumors such as Hurthle cell, tall cell, and insular variants of PTCs. FVPTCs can be distinguished on the molecular level from the conventional type by an inverse distribution of mutations affecting key molecules of the mitogen activated protein kinase (MAKP) pathway. Whereas *BRAF* mutations, commonly affecting codon 600 (V600E), are found in about 40 to 70 % of conventional PTCs, only 10 to 20 % of FVPTCs harbor this mutation [3,4]. In contrast, mutations in the highly related RAS genes, *HRAS*, *KRAS*, and *NRAS*, are identified only sporadically in conventional PTCs but in about 20% of FVPTCs [5]. Whereas a BRAF mutation represents a valuable target for molecular therapy in advanced solid tumors such like PTCs, valuable molecular targets in thyroid carcinomas bearing no *BRAF* mutation are less known [6]. Furthermore, RAS mutations are not considered as a sole key event of malignant transformation as they are frequently present in benign proliferative lesions of the thyroid such as follicular adenomas (FAs). We have identified RAS mutations in about 25 % of FAs which is consistent with findings of other studies [5,7]. It has beena subject of discussion as to what extent FAs or subtypes of FAs represent precursor lesions for FVPTCs [8]. Furthermore, it is of clinical relevance to unambiguously distinguish FAs from FVPTCs as FA lesions are cured by partial or (sub)total thyroidectomy whereas a substantial part of FVPTCs may have progressed further at time of first clinical treatment which requires clinical follow-up. Frequency of lymph node metastasis in FVPTC depends on predictive risk factors as multifocality and invasive behavior [9]. Criteria to establish a histopathological diagnosis of FVPTC include cytoplasmic or capsular invasion and nuclei that are ground glassed and/or endowed with abundant grooves [10]. A study using immunohistochemistry identified a panel of markers including HBME-1, CITED1, galectin-3, cytokeratin 19, and S100A4 that were able to distinguish FAs from FVPTCs [8]. Until now, only a few studies compared expression profiles between benign and malignant thyroid lesions [11-15]. All these studies included different types of benign or malignant follicular lesions which limits the comparison of the identified gene sets between the studies. Furthermore, as FVPTCs share histological features with both PTCs and follicular thyroid carcinomas (FTCs) a standardized diagnosis remains challenging [16,17]. We employed whole-transcript oligonucleotide microarrays to identify differentially expressed genes which could gain importance as molecular biomarkers that separate both follicular lesions on the molecular level. Normal thyroid specimens were included in expression profiling to serve as reference for normal expression levels. A limited number of samples within the comparison groups, like in our study, have been successfully used in other expression studies to generate differentially expressed gene sets in thyroid cancer [11,13,14].

## Methods

### Thyroid samples

We studied specimens from six FVPTCs, seven FAs, and seven normal thyroid (TN) samples that were derived from patients who were treated surgically in the period between February 2009 and April 2013 at the King Abdulaziz University Hospital (KAUH), Jeddah, and the King Faisal Specialist Hospital & Research Center (KFSH&RC), Jeddah. Normal thyroid specimens were derived from unaffected normal thyroid specimens. Diagnosis was established by an experienced oncologic pathologist (JM) according to established criteria [18,19]. DNA extraction of all samples and *BRAF* mutational screening for the 13 tumor samples was performed as described earlier [3]. This study was approved by the ethical review boards of KAUH (no. 358-10) and KFSH&RC (no. IRB2010-07).

### RNA and array processing

Total RNA was extracted from fresh tissue specimens using the Qiagen RNeasy Mini Kit (Qiagen, Hilden, Germany). Extraction protocol included an on-column DNAse treatment as recommended by the manufacturer. Quality of purified RNA was assessed using an Agilent 2100 Bioanalyzer (Agilent Technologies, Palo Alto, CA). RNA integrity number for all evaluated samples was at least 5.0. The NanoDrop ND-1000 spectrophotometer (NanoDrop Technologies, Wilmington, DE) was utilized to determine RNA concentration. Samples each consisting of 250 ng RNA were processed using the Ambion WT Expression Kit (Life Technologies, Austin, TX) and the GeneChip WT Terminal Labeling and Controls Kit (Affymetrix, Santa Clara, CA) according to the manufacturers` recommendations. In following processing steps the Affymetrix GeneChip Hybridization, Wash and Stain Kit was utilized. The hybridization mixtures containing each 5500 ng of cDNA were hybridized for 17 hrs to Affymetrix Human Gene 1.0 ST GeneChip arrays in a hybridization oven at 45°C under rotation (60 rpm). This array type interrogates with a set of 764,885 probes 36.079 annotated reference sequences (NCBI build 36). Subsequent to wash and staining, the arrays were scanned in the GeneChip Scanner 3000 7G. Probe cell intensity data (CEL files) were generated using the GeneChip Command Console Software (AGCC).

### Gene expression and alternative splicing analysis

CEL files were imported to Partek Genomics Suite version 6.6 (Partek Inc., MO) and a log-transformed data set of robust multi-chip averaged (RMA), background-adjusted, and normalized values generated using the RMA defaults settings as configured in the Advanced Import Option Tab. Quality of experiments was assessed on the basis of the QC metrics table and QC graphical report. Principal component analysis (PCA) was performed to assess quality as well as overall variance in gene expression between sample groups. Analysis of Variance (ANOVA) was applied to generate a list of differentially expressed genes and exons using a false discovery rate (FDR) < 0.05 and a fold change cut-off >2. FDR is the proportion of false positives among all positives. In Genomics Suite the step up method was implemented to control the FDR. Two dimensional average linkage hierarchical clustering was generated utilizing Spearman’s correlation as a similarity matrix on the set of differentially expressed genes (FVPTC *vs*. FA). To identify significant genes that intersect or non-intersect between differentially expressed groups, a Venn diagram was created from the Venn diagram menu tab by selecting the corresponding gene lists. For alternative splicing analysis, ANOVA was applied to identify probes with differentially expressed exons using FDR < 0.05 and a fold change cut-off > 2. The performance of Human Gene 1.0 ST arrays to detect alternative splicing events has been assessed [20]. The Partek Gene Ontology (GO) enrichment tool was used in the gene expression workflow to group significantly expressed genes (FVPTC *vs*. FA) into functional categories in the domains cellular components, molecular functions, and associated biological processes [21]. The GO enrichment score indicates the level of differential expression of genes in a functional category. The score is determined by utilizing a Fisher`s Exact test to compare within a GO domain the proportion of genes represented in a functional category to the proportion not presented in the functional category. The GO data resources are curated by the GO consortium in a controlled and structured vocabulary. The generated array data set complies with MIAME [22] and has been deposited at the NCBI’s Gene Expression Omnibus (GEO) under accession number GSE54958.

### Functional network and pathway analysis

The Ingenuity Pathways Analysis software (IPA) (Ingenuity Systems, Redwood City, CA) was employed to interpret our expression data in more detail. The main features of IPA are to provide biological insights into a data set by (i) mapping data to the Ingenuity knowledge base that contains IPA curated and GO information, (ii) translating data into diseases and biological functions, (iii) creating de novo molecular networks using algorithms based on biological information, (iv) and determining canonical pathways that are related to the data set. IPA results are sorted and ranked based on statistic scores. We initiated IPA by uploading the gene symbols as clone identifiers, *p*-values, and fold changes of the set of our differentially expressed genes. IPA workflow comprised core and functional analysis and structural categorization of the processed data. Network assembling was based on the connectivity of the differential expressed genes under the assumption the higher the connectivity of a gene the more significant it is. Non-connective areas were filled with genes from the IPA knowledge base. Canonical pathway analysis included an overlay analysis of predefined pathways with the gene list.

## Results

Using Affymetrix HuGene 1.0 ST arrays we compared expression profiles of 6 FVPTCs with 7 FAs and used 7 normal thyroid samples for reference expression. Mean age in both groups was 37 years and besides two male patients with an FA all other patients were females (Additional file 1). In FVPTCs, tumor stage I was revealed in four cases, stage III and stage IV in one case each. No *BRAF* mutation was identified in the mutational hotspot region of exon 15 in all 13 assayed tumors.

### Expression of FVPTC *vs*. FA

**Figure 1:**
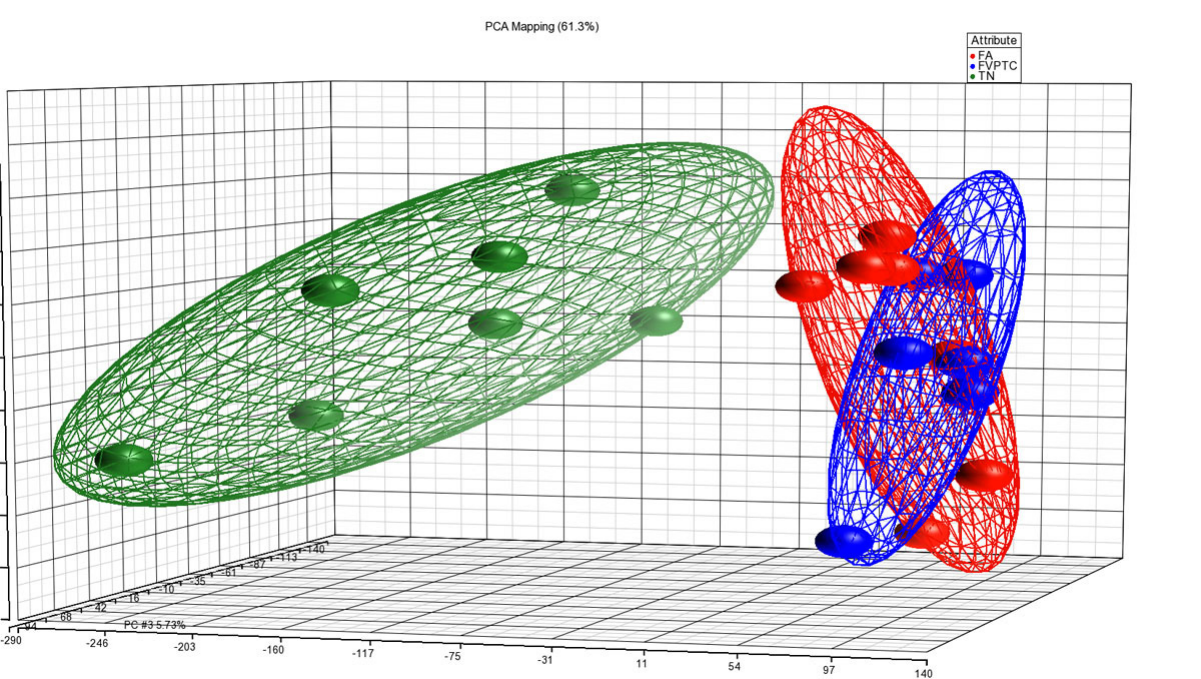
PCA scatter plot wherein each dot represents a sample with a group specific color. Distance between dots is a dimensional measure for the similarity of the expression profiles of the samples. Bounding ellipsoids are included for each group.

Three-D presentation of the PCA demonstrated that FAs and FVPTCs are clustering closely together as indicated by the bounding ellipsoids (Figure 1). We identified 55 transcripts that were differentially expressed between FAs and FVPTCs (*p*-value with FDR < 0.05 and fold change cut-off > 2) (Table 1). Forty genes were upregulated and 15 genes downregulated in FVPTCs compared to FAs. The most upregulated genes in FVPTCs include *GABA acid B receptor*, *2* (*GABBR2*), *neuronal cell adhesion molecule* (*NRCAM*), *extracellular matrix protein 1* (*ECM1*), *heparan sulfate 6-O-sulfotransferase 2* (*HS6ST2*), and *retinoid X receptor*, *gamma* (*RXRG*). The most significantly downregulated genes in FVPTCs include *interaction protein for cytohesin exchange factors 1* (*IPCEF1*), *G protein-coupled receptor 155* (*GPR155*), *Purkinje cell protein 4* (*PCP4*), *chondroitin sulfate N-acetylgalactosaminyltransferase 1* (*CSGALNACT1*), and *glutamate receptor interacting protein 1* (*GRIP1*). In the top five genes that were overexpressed in FVPTCs, the fold change (range, 4.09-18.36) was comparably higher than in the top five genes that were overexpressed in FAs (range, 2.47 - 6.48). Other genes, all overexpressed in FVPTCs, with a fold change ≥ 10 include *leucine-rich repeat* (*kinase 2*) (*LRRK2*), *odz*, *odd Oz/ten-m homolog 1* (*Drosophila*) (*ODZ1*), *lipase*, *member H* (*LIPH*), and *proteolipid protein 1* (*PLP1*).

**Table 1:** Fifty-five genes differentially expressed between FVPTCs and FAs and comparison with TN sample expression

Gene name	Gene symbol	FVPTC vs. FA		FVPTC vs. TN		FA vs. TN	
		
		FC^1^	*P* ^2^	FC^1^	*P*	FC^1^	*P*
gamma-aminobutyric acid (GABA) B receptor, 2	GABBR2	7.09	1.06E-07	8.51	2.99E-08	1.20	4.10E-01

neuronal cell adhesion molecule	NRCAM	4.09	1.64E-07	4.06	1.76E-07	-1.01	9.64E-01

extracellular matrix protein 1	ECM1	18.36	5.77E-07	15.49	1.28E-06	-1.19	6.44E-01

heparan sulfate 6-O-sulfotransferase 2	HS6ST2	5.07	8.60E-07	3.77	1.11E-05	-1.35	1.71E-01

retinoid X receptor, gamma	RXRG	13.16	8.69E-07	15.64	3.64E-07	1.19	6.08E-01

insulin-like growth factor 2 mRNA binding protein 2	IGF2BP2	5.88	1.10E-06	4.90	4.45E-06	-1.20	4.40E-01

cellular retinoic acid binding protein 2	CRABP2	4.48	1.18E-06	4.20	2.07E-06	-1.07	7.50E-01

high mobility group AT-hook 2	HMGA2	5.15	1.33E-06	3.17	8.64E-05	-1.62	3.91E-02

interaction protein for cytohesin exchange factors 1	IPCEF1	-6.48	1.39E-06	-3.55	1.32E-04	1.82	2.70E-02

G protein-coupled receptor 155	GPR155	-2.83	2.09E-06	-1.31	8.64E-02	2.16	4.76E-05

Purkinje cell protein 4	PCP4	-2.47	2.17E-06	-1.50	5.96E-03	1.65	9.10E-04

dipeptidyl-peptidase 10 (non-functional)	DPP10	3.85	2.22E-06	1.99	2.34E-03	-1.93	2.49E-03

low density lipoprotein receptor-related protein 4	LRP4	8.23	3.70E-06	13.56	2.22E-07	1.65	1.16E-01

chondroitin sulfate N-acetylgalactosaminyltransferase 1	CSGALNACT1	-3.34	4.06E-06	-1.13	5.01E-01	2.95	9.50E-06

fibronectin type III domain containing 4	FNDC4	3.65	5.75E-06	2.48	2.85E-04	-1.47	6.15E-02

leucine-rich repeat kinase 2	LRRK2	20.97	6.06E-06	31.24	1.27E-06	1.49	3.92E-01

odz, odd Oz/ten-m homolog 1 (Drosophila)	ODZ1	13.95	6.87E-06	13.63	7.66E-06	-1.02	9.54E-01

lipase, member H	LIPH	18.83	7.39E-06	20.52	5.19E-06	1.09	8.49E-01

glutamate receptor interacting protein 1	GRIP1	-2.52	7.61E-06	-1.24	1.66E-01	2.04	9.33E-05

slit homolog 1 (Drosophila)	SLIT1	6.12	8.81E-06	9.01	7.51E-07	1.47	1.83E-01

protocadherin 7	PCDH7	3.46	8.83E-06	2.96	4.20E-05	-1.17	4.26E-01

proteolipid protein 1	PLP1	10.00	8.88E-06	4.92	4.63E-04	-2.03	6.14E-02

SOGA family member 3	SOGA3	2.54	9.38E-06	2.44	1.59E-05	-1.04	7.81E-01

patched domain containing 4	PTCHD4	9.30	1.03E-05	3.03	7.04E-03	-3.07	4.90E-03

UDP-N-acetyl-alpha-D-galactosamine:polypeptide N-acetylgalactosamine	GALNT7	3.57	1.04E-05	8.03	1.35E-08	2.25	7.59E-04

glutaminyl-peptide cyclotransferase	QPCT	6.05	1.18E-05	5.68	1.79E-05	-1.06	8.27E-01

transcription factor CP2-like 1	TFCP2L1	-4.40	1.42E-05	-1.80	2.95E-02	2.45	1.51E-03

cell wall biogenesis 43 C-terminal homolog (S. cerevisiae)	CWH43	-4.73	1.47E-05	-2.32	4.77E-03	2.04	1.10E-02

pleiomorphic adenoma gene 1	PLAG1	5.46	1.47E-05	3.88	1.75E-04	-1.41	2.26E-01

UDP-galactose-4-epimerase	GALE	2.74	1.52E-05	2.39	8.06E-05	-1.15	4.04E-01

neurotrophic tyrosine kinase, receptor, type 3	NTRK3	3.64	1.75E-05	2.68	3.21E-04	-1.36	1.61E-01

v-kit Hardy-Zuckerman 4 feline sarcoma viral oncogene homolog	KIT	-4.84	1.93E-05	-1.47	1.72E-01	3.30	2.53E-04

acid phosphatase, prostate	ACPP	2.41	2.59E-0	2.48	1.72E-05	1.03	8.34E-01

diacylglycerol kinase, iota	DGKI	-3.53	3.16E-05	-1.88	1.19E-02	1.87	9.87E-03

vitamin D (1,25- dihydroxyvitamin D3) receptor	VDR	2.62	3.26E-05	2.63	3.09E-05	1.00	9.79E-01

family with sequence similarity 70, member A	FAM70A	2.74	3.30E-05	1.05	7.91E-01	-2.61	3.66E-05

uncharacterized LOC100129434	LOC100129434	-2.60	3.63E-05	-1.37	8.73E-02	1.90	1.22E-03

spermatogenesis associated 18	SPATA18	2.70	4.41E-05	1.95	1.93E-03	-1.38	8.18E-02

retrotransposon gag domain containing 4	RGAG4	3.13	4.80E-05	4.19	3.16E-06	1.34	1.66E-01

zinc finger protein 521	ZNF521	2.72	5.44E-05	4.00	1.03E-06	1.47	4.63E-02

pleckstrin and Sec7 domain containing 3	PSD3	3.50	5.69E-05	8.64	5.67E-08	2.47	9.62E-04

synapse differentiation inducing 1	SYNDIG1	-2.67	5.72E-05	-1.47	5.38E-02	1.82	3.60E-03

LIM domain only 3 (rhombotin-like 2)	LMO3	2.66	5.86E-05	5.59	4.25E-08	2.10	6.13E-04

adhesion molecule with Ig-like domain 2	AMIGO2	2.87	5.90E-05	2.51	2.35E-04	-1.14	5.02E-01

RNA binding protein with multiple splicing 2	RBPMS2	-2.73	6.11E-05	1.01	9.51E-01	2.76	3.45E-05

integrin, alpha 2 (CD49B, alpha 2 subunit of VLA-2 receptor)	ITGA2	2.72	6.37E-05	5.15	1.30E-07	1.89	2.75E-03

sodium channel, voltage-gated, type IV, alpha subunit	SCN4A	2.29	6.51E-05	2.70	8.15E-06	1.18	2.95E-01

neuro-oncological ventral antigen 1	NOVA1	3.86	7.79E-05	2.49	2.83E-03	-1.55	9.88E-02

SMAD family member 9	SMAD9	-3.03	7.91E-05	-1.18	4.60E-01	2.57	2.66E-04

Sciellin	SCEL	5.85	8.10E-05	6.23	5.50E-05	1.07	8.48E-01

SLC26A8	SLC26A8	2.71	8.28E-05	1.45	7.47E-02	-1.87	3.65E-03

serine-rich and transmembrane domain containing 1	SERTM1	-5.94	8.38E-05	-1.58	2.05E-01	3.76	9.96E-04

calpain 3 (p93)	CAPN3	2.23	8.41E-05	2.93	2.60E-06	1.32	8.34E-02

immunoglobulin superfamily, member 1	IGSF1	8.63	8.51E-05	9.28	5.97E-05	1.07	8.60E-01

primary ciliary dyskinesia protein 1	PCDP1	-2.17	8.74E-05	-1.23	1.91E-01	1.77	1.15E-03

^1^FC, fold change;^2^p-value with FDR < 0.05 and fold change cutoff > 2;-, downregulated in FVPTCs vs. FAs, downregulated in FVPTCs vs. TN, and downregulated in FAs vs. TN.

**Figure 2:**
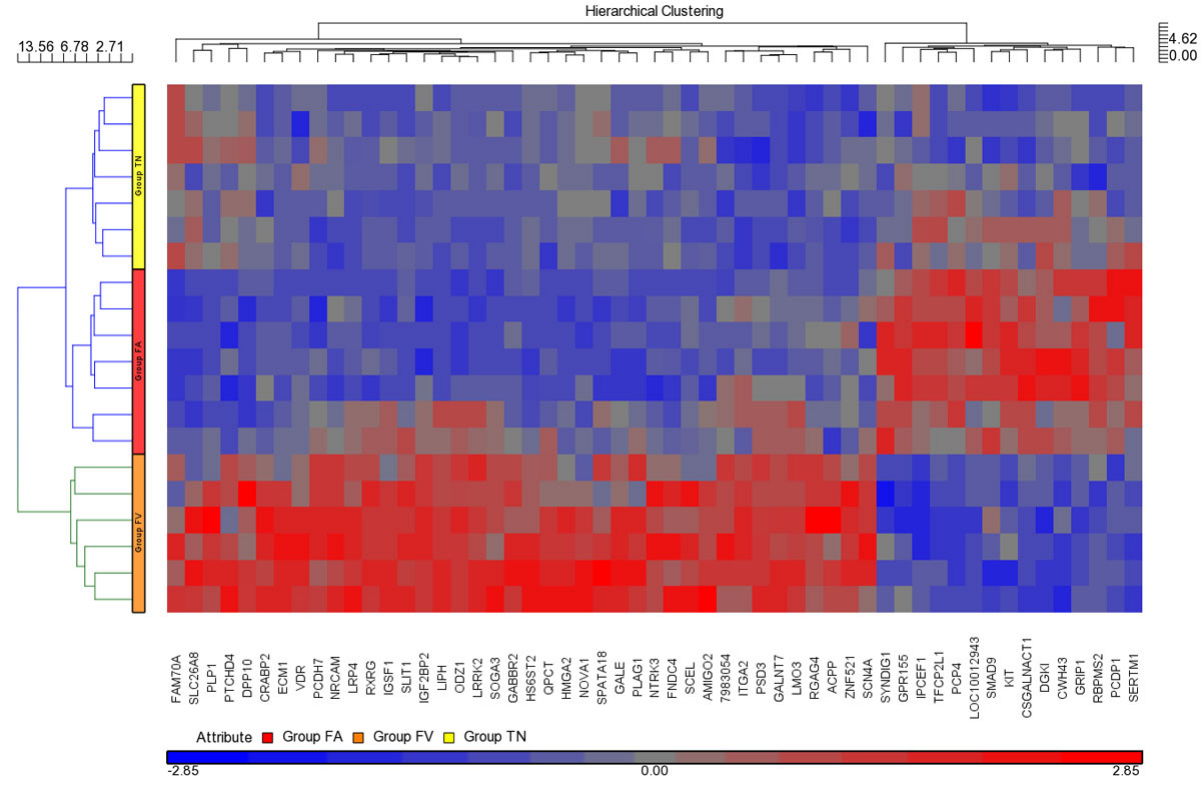
Hierarchical cluster analysis of 55 genes which were differentially expressed (FDR *p*-value ≤ 0.05 and fold change > 2.0) between FAs and FVPTCs. Gene expression of TN samples is included in analysis. Color scheme, red for comparably higher and blue for comparably lower expression.

Hierarchical cluster analysis displays the diverging expression profiles of the 55 differentially expressed genes between FAs and FVPTCs (Figure 2). TN samples were included to provide expression values of normal thyroid tissue. Comparison of either FVPTCs or FAs with TN samples revealed 12280 and 11706 differentially expressed transcripts, respectively. The number of intersecting and non-intersecting genes between the three sets of differentially expressed genes is projected into a Venn diagram illustrating the molecular relationships between the groups (Additional file 2).

### Enrichment score analysis

An enrichment analysis for functional categories in the GO domains cellular component, molecular process, and biological functions was performed on the set of 55 differentially expressed genes (Additional file 3). The most dominant functional categories in the cellular component domain were membrane part (enrichment score 4.90), synapse part (5.73), and membrane (6.16). In the molecular function domain, the prevalent categories consisted of molecular transducer activity (2.72), binding (2.72), and nucleic acid binding transcription factor activity (2.87) whereas in the biological process domain, the dominant categories included localization (5.80), single-organism process (6.03), and developmental process (10.23).

### Exon splicing

Statistical exon splicing analysis identified 62 genes for which at least one exon was significantly upregulated and 25 genes for which at least one exon was significantly downregulated in FVPTCs compared to FAs (Additional file 4). This gene list highly overlaps with the list of differentially expressed genes (Table 1) as 52 of the genes were identical in both sets. An example for an exon splicing event is provided for *HS6ST2* (Additional file 5).

### Network and pathway analysis

**Figure 3:**
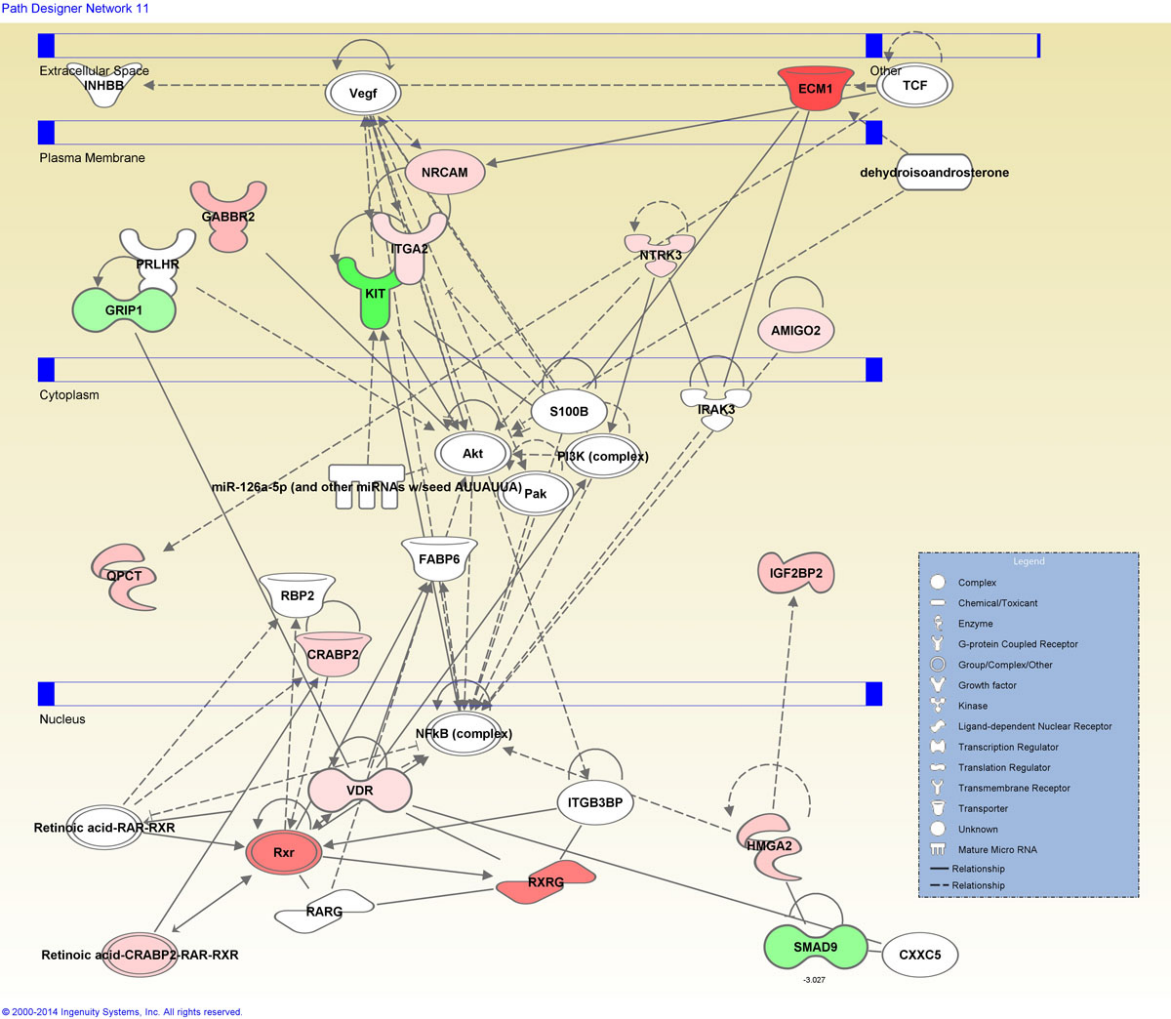
Molecular network analysis based on the set of differentially expressed genes that are known to interact directly or, via intermediate molecules, indirectly. Molecules like ECM1, SMAD9, KIT, VDR, NRCAM, and RXRG are involved in multiple interactions. Color scheme, red for comparably higher and green for comparably lower expression in FVPTCs *vs*. FAs.

Using IPA, a network was assembled based on those connective relationships that were identified in our differentially expressed genes (Figure 3). Upregulated network molecules in FVPTCs include molecules such as ECM1, vitamin D (1,25-dihydroxyvitamin D3) receptor (VDR), NRCAM, and RXRG and downregulated molecules in FVPTCs include SMAD family member 9 (SMAD9), v-kit Hardy-Zuckerman 4 feline sarcoma viral oncogene homolog (KIT), and GRIP1. The most significantly associated canonical pathway related to the differentially expressed gene set was thyroid cancer signaling (*p* = 5.58x10^-3^).

## Discussion

With our gene expression study we were aiming to identify molecular features that differentiate FVPTC from FA. We detected a set of significantly differentially expressed genes that vary from studies comparing FAs with FTCs, FVPTCs, or PTCs [11,13,14,23,24]. Oligonucleotide microarray studies that compared either PTCs/FTCs/FVPTCs with FAs/hyperplastic lesions [12] or FTCs with FAs [14] detected 133 and 105 differentially expressed genes, respectively; however, virtually no genes of these studies were shared with our 55 differentially expressed genes.

### Top genes upregulated in FVPTC

Among the most overexpressed genes in FVPTCs in our study were *GABBR2*, *NRCAM*, *ECM1*, *HS6ST2*, *RXRG*, *LRRK2*, *ODZ1*, *LIPH*, and *PLP1*. GABBR2 is a component of the heterodimeric G protein-coupled receptor for GABA. Overexpression of *GABBR2* has been identified in pediatric post-Chernobyl PTCs compared to sporadic adult PTCs [25]. NRCAM is a member of the neuronal cell adhesion molecules. Prominent overexpression of NRCAM has been detected in PTCs compared to matched normal tissue by RT-PCR and immunohistochemistry which led to the suggestion that the molecule is involved in PTC pathogenesis [26]. ECM1 is a soluble molecule that is implicated in angiogenesis and endochondral bone development. Upregulation of *ECM1* has been revealed in malignant compared to benign thyroid neoplasms and it has been proven to be useful as a diagnostic marker for thyroid malignancy in fine needle aspiration biopsies [27]. HS6ST2 is a membrane and extracellular matrix associated molecule. Gene silencing of *HS6ST2* in pancreatic cells resulted in inhibition of cell progression [28]. RXRG is a member of the retinoid X receptors and forms heterodimers with retinoic acid, VDR, and the thyroid hormone receptor. Significant overexpression of *RXRG* has been detected in PTCs compared normal thyroid tissue and the receptor proved to be useful in a classifier set consisting of 19 genes to accurately discriminate PTCs from normal thyroid tissue [29]. A biostatistics and microarray survey in PTC and papillary renal cell carcinomas detected concomitant overxpression of *LRRK2* and the *MET* proto-oncogene [30]. Knockdown of *LRRK2* in cell culture experiments minimized MET phosphorylation and impaired MET downstream signaling to STAT3 and mTOR resulting in cell cycle arrest. *LRRK2* was considerably higher expressed in our FVPTCs compared to FAs (fold change, 20.97) underscoring the known relevance of the MET pathway in thyroid malignancies [31]. ODZ1 belongs to a conserved group of signaling molecules acting as transmembrane receptors and with their intracellular domain as transcriptional factors [32]. An oligonucleotide array study detected upregulation of *ODZ1* in PTCs compared to matched normal thyroid samples and the results were confirmed by RT-PCR [33]. The PLP1 protein is a major myelin component and a marker for oligodendrocytes. Functional involvement of PLP1 in thyroid cancer is virtually unknown; however, in glioblastoma multiforme, *PLP1* expression correlated with the extent of the oligodendroma component [34]. LIPH is a secreted enzyme that hydrolysis phosphatidic acid into free fatty acid and lysophosphatidic acid which is known as a ligand for a number of G protein-coupled receptors and implicated in receptor signaling in cancer [35]. Based on a microarray data survey, *LIPH* has been recently discovered as a putative biomarker for lung adenocarcinomas and bronchioloalveolar carcinomas [36]. LIPH was also found to be higher expressed in the serum of lung cancer patients.

### Top genes downregulated in FVPTC

Among the most downregulated genes in FVPTCs compared to FAs were *IPCEF1*, *GPR155*, *PCP4*, *CSGALNACT1*, and *GRIP1*. IPCEF1 has been identified as the C-terminal part of a protein in which CNK3 represents the N-terminal part [37]. *In vitro* experiments have demonstrated that knockdown of *CNK3*/*IPEF1* leads to inhibition of hepatocyte growth factor induced cell migration. *GPR155* is an integral membrane G protein-coupled receptor harboring an auxin efflux carrier domain, a pleckstrin/G protein-interacting region, and a winged helix repressor DNA-binding domain; however, the function of the receptor is barely known [38]. *PCP4* codes for a sodium-dependent neurotransmitter. Upregulation of *PCP4* was detected in cerebral tissue from rats that were hyperthyroidic through triiodothyronine application [39]. CSGALNACT1 is a critical glycosyltransferase of chondroitin sulfate biosynthesis. Highest RNA expression among different tissue types tested was identified in the thyroid and placenta and statistical network reconstruction on expression data of multiple myeloma identified that high expression of *CSGALNACT1* was associated with favorable prognosis [40,41]. *GRIP1*, also known as *nuclear receptor coactivator 2* (*NCoA-2*) encodes a transcriptional co-activator of steroid, thyroid, retinoid, and vitamin D receptors including thyroid hormone receptor alpha [42,43]. GRIP1 assists these nuclear receptors in promoter-driven DNA expression.

### Network genes

Network analysis displayed interactions wherein approximately one third of our set of 55 differentially expressed genes participated either in the extracellular space, transmembrane domain or intracellular compartments (Figure 3). Involved in multiple interactions were a number of molecules including ECM1, VDR, SMAD9, KIT, NRCAM, and RXRG. VDR is involved in gene activation through heterodimerization with the retinoid X receptor and subsequent binding to vitamin D responsive elements [44]. The functional role of the retinoid X receptor as a heterodimerization partner of the thyroid hormone receptor in hormone signaling pathways depends on the cellular context [45]. Further investigations should be envisaged to address the functional role of RXRG, VDR, and the thyroid hormone receptor in thyroid cancer, especially in FVPTCs. SMAD9 is a member of the receptor SMAD family that is involved in the canonical bone morphogenetic pathway. SMAD9 is known to interact with high-mobility group AT-hook 2 (HMGA2) for which a higher expression has been detected in a quantitative RT-PCR assay in FVPTCs and PTCs compared to FAs [46]. *KIT* encodes a receptor tyrosine kinase type III and activation mutations are associated with a number of tumor types; however, decreased *KIT* expression has been identified in malignant thyroid lesions compared to benign lesions [47] which is consistent with our findings.

### Nucleic acid binding factors

IGF2BP2 is a member of the insulin-like growth factor pathway and it has been shown that its expression correlates with tumor progression in head and neck squamous cell carcinoma patients [48]. Knockdown of *HGMA2* in embryonic rhabdomyosarcoma cells resulted in severe downregulation of *IGF2BP2* and knockdown of either *HGMA2 or IGF2BP2* led to reduced protein levels encoded by the *neuroblastoma RAS viral* (*v-ras*) *oncogene homolog* (*NRAS*) [49]. Furthermore, binding of IGF2BP2 to the 3`UTR of *NRAS* resulted in increased stability of the *NRAS* mRNA and enhanced NRAS protein levels. As gain-of-function mutations in *NRAS* are found in a minority of FVPTCs [5], overexpression of *IGF2BP2* would be an intriguing alternative that leads to enhanced NRAS signalling in thyroid cancer with follicular histology. CRABP2 is known as a co-activator of retinoid acid and retinoid acid receptor signaling [50]. Hypermethylation of CpG islands in the *CRABP2* promoter was associated with its downregulation in PTC compared to normal thyroid tissue [51]. We observed a higher expression of *CRABP2* in FVPTCs compared to FAs but of notice, *CRABP2* was lower expressed (fold change, 1.1) in our FAs compared to TN samples. TFCP2L1 is a *crystallin*, *alpha B* (*CRYAB*) gene promoter interacting transcription factor and lower expression of *TFCP2L1* and *CRYAB* was revealed in anaplastic thyroid carcinomas compared to benign goiters [52]. Lower expression of *TFCP2L1* was also detected in PTCs compared to matched normal thyroid samples in a combined microarray/RT-PCR study [53]*.* The zinc finger factor PLAG1 is a transcriptional activator of a number of genes including *insulin-like growth factor II* and the *cytokine-like factor 1*[54]*.* An RT-PCR analysis of a number of candidate genes for differentiating benign and malignant thyroid lesions detected concordant higher expression of *PLAG1* and *HMGA2* in FVPTCs, and to a lesser extent in FTCs, compared to other thyroid malignancies (PTCs and a Hurthle cell carcinoma) or benign forms of proliferative thyroid lesions [15]. A strong correlation of *PLAG1* and *HMGA2* expression was also detected in a number of thyroid carcinomas and uterine leiomyomas and cell culture experiments led to the conclusion that HMGA2 is an upstream activator of *PLAG1*[46]. Expression of zinc finger transcription factor ZFN521 is known to be required for embryonic stem cell differentiation from the epiblast state into neuroectodermal progenitor cells [55]. Furthermore, cell culture assays indicated that enhanced expression of ZFN521 is associated with reduced recognition of tumor cells by natural killer cells [56]. Downregulation of *LMO3* in radiation-related thyroid carcinomas in comparison to matched normal thyroid samples has been detected in a microarray/RT-PCR study conducted on individuals who received iodine-131 doses as a consequence of the Chernobyl accident [57]. In contrast, overexpression of *LMO3* has been demonstrated as a marker for development and progression of neuroblastoma and furthermore, cell culture experiments indicated that LMO3 is a p53 co-suppressor [58]. In lung adenocarcinomas bearing a *NK2 homeobox 1* (*NKX2-1*) amplification, the oncogenic capacity of LMO3 as a transcription regulator downstream of NKX2-1 has been demonstrated [59]. The *neuro-oncological ventral antigen 1* (*NOVA1*) encodes an alternative splicing factor and is transcriptionally subject to A-to-I editing [60]. High expression of the NOVA1 RNA-binding protein has been found to be associated with unfavorable prognosis in hepatocellular carcinoma [61].

In summary, our expression profile study identified a set of candidate genes which are implicated in molecular mechanisms that differentiate FAs from FVPTCs and thus, add to our knowledge of the tumor biology of these histopathologically related, but clinically distinct entities. Further studies have to address the question to what extent the identified genes are solely discriminators of histopathological interest or have functional implication in a not yet defined transition process from FA to FVPTC.

## List of abbreviations

FA: follicular adenoma; FTC: follicular variant of PTC; FVPTC or FV: follicular variant of PTC; PTC: papillary thyroid carcinoma; TN: normal thyroid.

## Competing interests

The authors declare that they have no competing interests.

## Authors’ contributions

JM and MHQ made substantial contributions to conception and design of the study. IB, NB, and OS processed expression arrays and were involved in data interpretation. AJ, AAM, KG and OAH were responsible for surgeries, and with ZM had oversight of clinical databases and contributed to the conception and design of the study. JM, HA, AM performed histological examinations. SK and HJS performed data analysis. HJS had general oversight of the study. HJS, JM, and MHQ interpreted data and drafted the manuscript. All authors read and approved the final manuscript

## Supplementary Material

Additional file 1 Demographic and clinicopathological features of FA and FVPTC cases. Click here for file

Additional file 2 A Venn diagram visualizing the number of differentially expressed genes that intersect or non-intersect between the comparison groups FVPTC *vs*. FA, FA *vs*. TN, and FVPTC vs. TN. Of the 55 differentially expressed genes in FVPTC *vs*. FA, nine were only differentially expressed within this comparison group whereas other 8 genes intersect with FA *vs*. TN and another 33 genes intersect with FVPTC *vs*. TN. Five differentially expressed genes were shared among all three groups. Click here for file

Additional file 3 GO enrichmment analysis for the 55 genes that were differentially expressed between FVPTC and FA. The functional categories are ranked according to their *p*-values. (A) In the cellular component domain, the categories membrane part (4.90), synapse part (5.73), and membrane (6.16) were prevalent. (B) In the molecular function domain, the prevalent categories were molecular transducer activity (2.72), binding (2.72), and nucleic acid binding transcription factor activity (2.87). (C) In the biological process domain, the dominant categories were localization (5.80), single-organism process (6.03), and developmental process (10.23). Click here for file

Additional file 4 **Eighty-seven genes with exons differentially expressed between FVPTCs and FAs.** This is an unfiltered gene list based on statistics measures and may include genes where, e.g. different exon expression levels were caused by RNA degradation. Click here for file

Additional file 5 **Exon splicing in *heparan sulfate 6-O-sulfotransferase 2* (*HS6ST2*). The exons of the gene are interrogated with nine oligonucleotide probes of which five are highly overexpressed in FVPTCs compared to FAs and TN samples. Exon splicing events in *HS6ST2* conferring different properties are known**[62]. Click here for file

## Declarations

Publication charges for this article have been funded by the Center of Excellence in Genomic Medicine Research (CEGMR), King Abdulaziz University, Jeddah, Kingdom of Saudi Arabia.

This article has been published as part of *BMC Genomics* Volume 16 Supplement 1, 2015: Selected articles from the 2nd International Genomic Medical Conference (IGMC 2013): Genomics. The full contents of the supplement are available online at http://www.biomedcentral.com/bmcgenomics/supplements/16/S1
