# Supplementary material for: Comparison of microarray expression profiles between follicular variant of papillary thyroid carcinomas and follicular adenomas of the thyroid
Source: BMC Genomics. 2015 Jan 15;16(Suppl 1):S7. doi: 10.1186/1471-2164-16-S1-S7 (PMC4315165; doi:10.1186/1471-2164-16-S1-S7)
Supplement: Additional file 1 — Demographic and clinicopathological features of FA and FVPTC cases. [file 1471-2164-16-S1-S7-S1.docx]

| **Additional file 1: Demographic and clinicopathological features of FA and FVPTC cases** | | | | | | |
| --- | --- | --- | --- | --- | --- | --- |
| **Case** | **Histology** | **Age (year)** | **Gender** | **Tumor size**  **(cm)** | **Stage** | **BRAF mutation** |
| TM-110-11 | Hurthle cell FA | 35 | M | 1 |  | neg |
| TM-116-12 | FA | 26 | F | 4 |  | neg |
| TM-214-09 | FA | 51 | F | 1 |  | neg |
| TM-269-11 | FA | 34 | F | 2.7 |  | neg |
| TM-601-11 | Hurthle cell FA | 30 | F | 2 |  | neg |
| TM-1163-11 | FA | 55 | F | 2 |  | neg |
| TM-2647-10 | FA | 28 | M | 2.3 |  | neg |
| TM-134-10 | FVPTC | 13 | F | 0.6 | I | neg |
| TM-301-12 | FVPTC | 51 | F | 5.5 | III | neg |
| TM-557-13 | FVPTC | 30 | F | 1.5 | I | neg |
| TM-989-11 | FVPTC | 71 | F | 8.5 | IVC | neg |
| TM-1188-12 | FVPTC | 34 | F | 5.0 | I | neg |
| TM-2646-10 | FVPTC | 25 | F | 7.0 | I | neg |

FA, follicular adenoma; FVPTC, follicular variant of papillary thyroid carcinoma; M, male; F, female;neg, negative.
