# Supplementary figures and images for: Comparison of microarray expression profiles between follicular variant of papillary thyroid carcinomas and follicular adenomas of the thyroid
Source: BMC Genomics. 2015 Jan 15;16(Suppl 1):S7. doi: 10.1186/1471-2164-16-S1-S7 (PMC4315165; doi:10.1186/1471-2164-16-S1-S7)

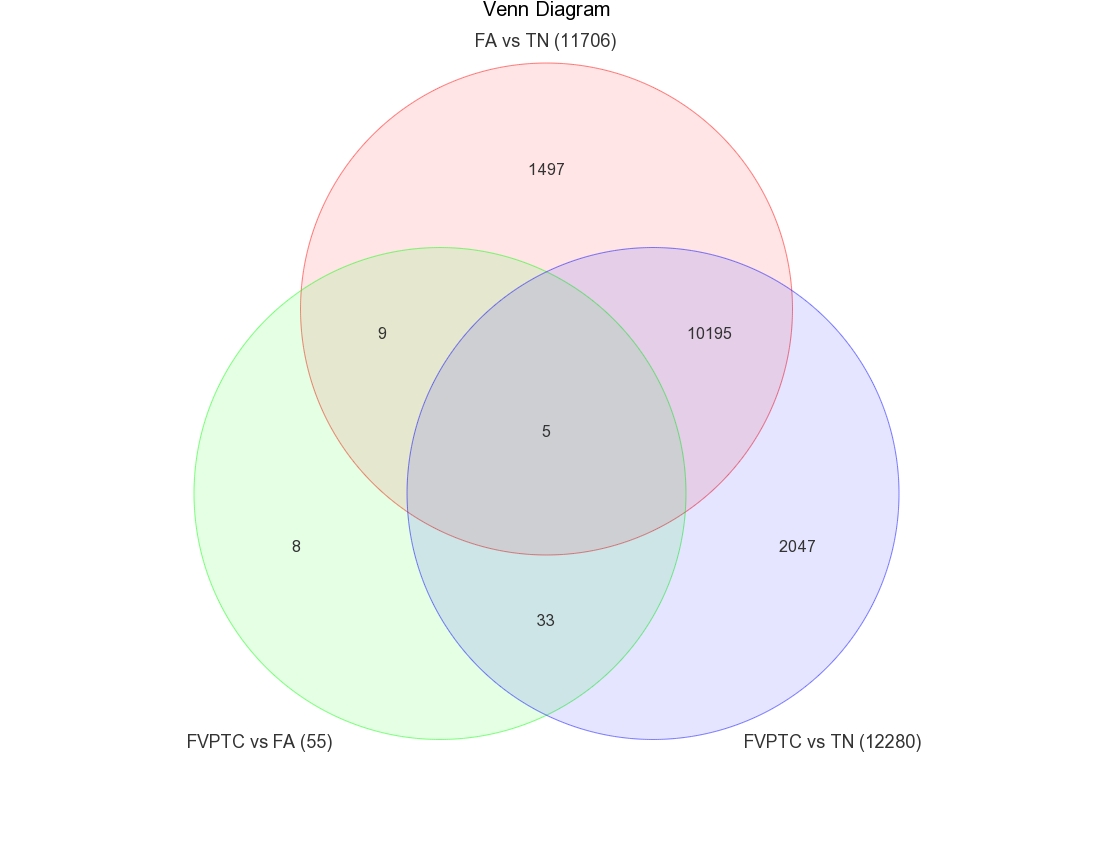

Supplement: Additional file 2 — A Venn diagram visualizing the number of differentially expressed genes that intersect or non-intersect between the comparison groups FVPTC vs. FA, FA vs. TN, and FVPTC vs. TN. Of the 55 differentially expressed genes in FVPTC vs. FA, nine were only differentially expressed within this comparison group whereas other 8 genes intersect with FA vs. TN and another 33 genes intersect with FVPTC vs. TN. Five differentially expressed genes were shared among all three groups. [file 1471-2164-16-S1-S7-S2.jpeg]

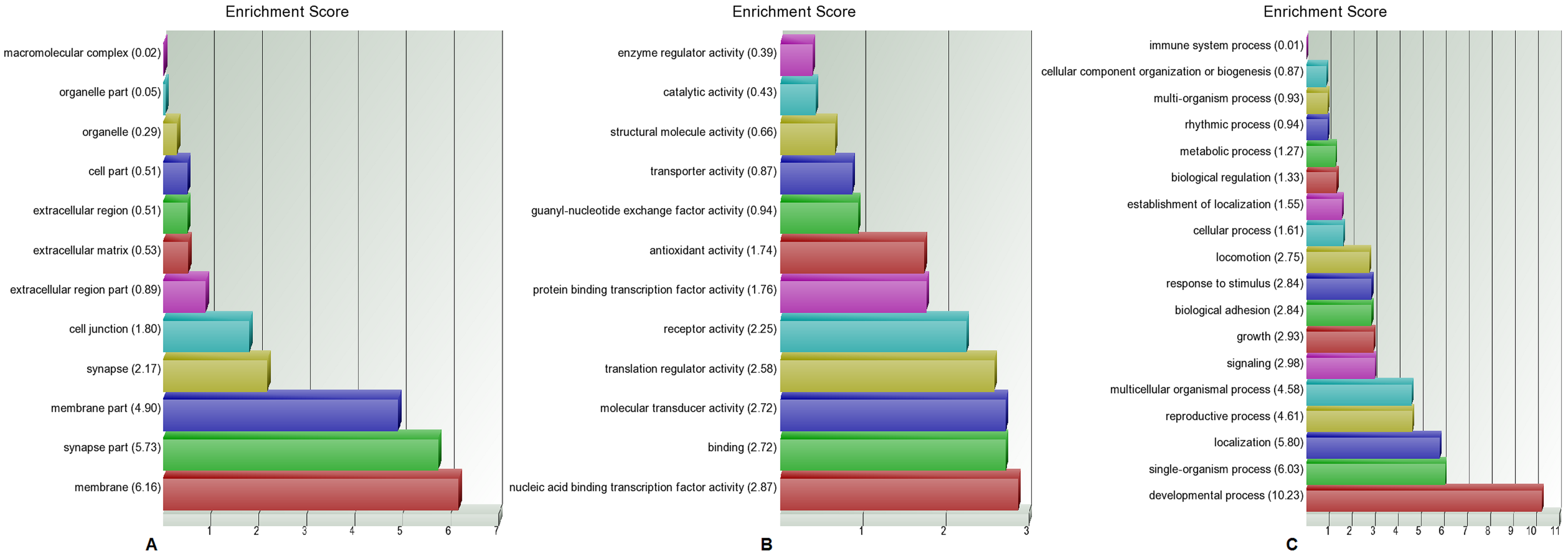

Supplement: Additional file 3 — GO enrichmment analysis for the 55 genes that were differentially expressed between FVPTC and FA. The functional categories are ranked according to their p-values. (A) In the cellular component domain, the categories membrane part (4.90), synapse part (5.73), and membrane (6.16) were prevalent. (B) In the molecular function domain, the prevalent categories were molecular transducer activity (2.72), binding (2.72), and nucleic acid binding transcription factor activity (2.87). (C) In the biological process domain, the dominant categories were localization (5.80), single-organism process (6.03), and developmental process (10.23). [file 1471-2164-16-S1-S7-S3.tif]

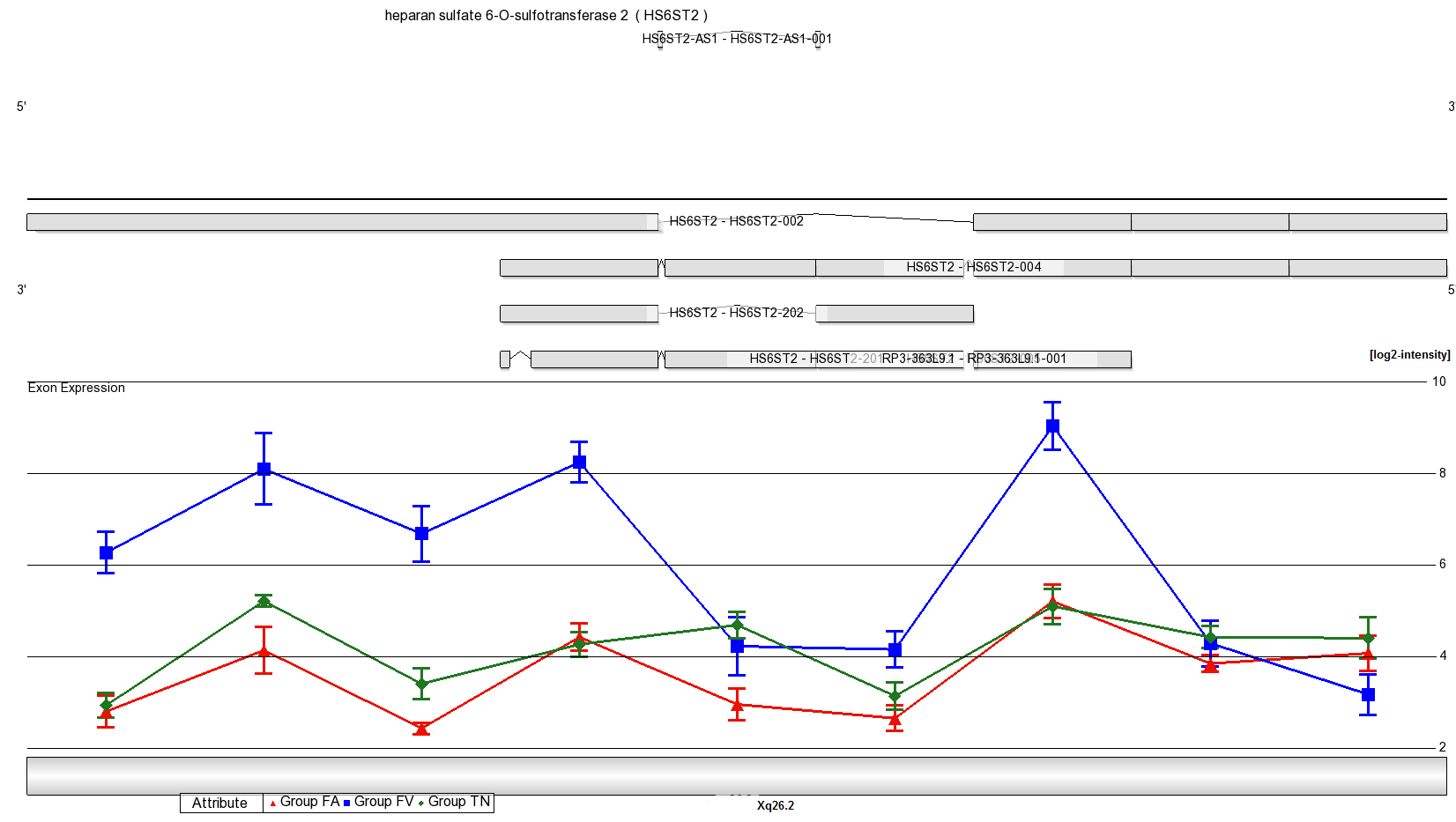

Supplement: Additional file 5 — Exon splicing in heparan sulfate 6-O-sulfotransferase 2 (HS6ST2). The exons of the gene are interrogated with nine oligonucleotide probes of which five are highly overexpressed in FVPTCs compared to FAs and TN samples. Exon splicing events in HS6ST2 conferring different properties are known[62]. [file 1471-2164-16-S1-S7-S5.tif]
