# Supplementary material for: Comparison of microarray expression profiles between follicular variant of papillary thyroid carcinomas and follicular adenomas of the thyroid
Source: BMC Genomics. 2015 Jan 15;16(Suppl 1):S7. doi: 10.1186/1471-2164-16-S1-S7 (PMC4315165; doi:10.1186/1471-2164-16-S1-S7)
Supplement: Additional file 4 — Eighty-seven genes with exons differentially expressed between FVPTCs and FAs. This is an unfiltered gene list based on statistics measures and may include genes where, e.g. different exon expression levels were caused by RNA degradation. [file 1471-2164-16-S1-S7-S4.docx]

**Additional file 4: Eighty-seven genes with exons differentially expressed between FVPTCs and FAs**

| **Gene name** | **Gene symbol** | **FVPTC vs. FA** | | **DEG^3^** |
| --- | --- | --- | --- | --- |
|  |  | **FC^1^** | ***P*^2^** |  |
| dipeptidyl-peptidase 10 (non-functional) | DPP10 | 4.73 | 7.5E-08 | 1 |
| cellular retinoic acid binding protein 2 | CRABP2 | 4.75 | 1.2E-07 | 1 |
| neurotrophic tyrosine kinase, receptor, type 3 | NTRK3 | 4.21 | 1.8E-07 | 1 |
| neuronal cell adhesion molecule | NRCAM | 3.81 | 4.5E-07 | 1 |
| gamma-aminobutyric acid (GABA) B receptor, 2 | GABBR2 | 6.05 | 4.6E-07 | 1 |
| insulin-like growth factor 2 mRNA binding protein 2 | IGF2BP2 | 6.39 | 7.5E-07 | 1 |
| G protein-coupled receptor 155 | GPR155 | -2.83 | 1.1E-06 | 1 |
| diacylglycerol kinase, iota | DGKI | -3.88 | 1.5E-06 | 1 |
| glutamate receptor interacting protein 1 | GRIP1 | -2.59 | 1.7E-06 | 1 |
| extracellular matrix protein 1 | ECM1 | 12.80 | 1.7E-06 | 1 |
| heparan sulfate 6-O-sulfotransferase 2 | HS6ST2 | 4.67 | 1.9E-06 | 1 |
| EDAR-associated death domain | EDARADD | -2.36 | 2.1E-06 | 0 |
| interaction protein for cytohesin exchange factors 1 | IPCEF1 | -6.34 | 2.4E-06 | 1 |
| high mobility group AT-hook 2 | HMGA2 | 5.16 | 2.6E-06 | 1 |
| Purkinje cell protein 4 | PCP4 | -2.14 | 3.0E-06 | 1 |
| retinoid X receptor, gamma | RXRG | 11.57 | 3.1E-06 | 1 |
| low density lipoprotein receptor-related protein 4 | LRP4 | 7.38 | 4.0E-06 | 1 |
| chondroitin sulfate N-acetylgalactosaminyltransferase 1 | CSGALNACT1 | -3.25 | 4.4E-06 | 1 |
| fibronectin type III domain containing 4 | FNDC4 | 3.34 | 5.4E-06 | 1 |
| deleted in lung and esophageal cancer 1 | DLEC1 | 2.08 | 6.7E-06 | 0 |
| odz, odd Oz/ten-m homolog 1 (Drosophila) | ODZ1 | 14.64 | 8.2E-06 | 1 |
| proteolipid protein 1 | PLP1 | 9.15 | 8.5E-06 | 1 |
| leucine-rich repeat kinase 2 | LRRK2 | 20.67 | 8.6E-06 | 1 |
| v-kit Hardy-Zuckerman 4 feline sarcoma viral oncogene homolog | KIT | -5.55 | 9.5E-06 | 1 |
| cell wall biogenesis 43 C-terminal homolog (S. cerevisiae) | CWH43 | -5.26 | 9.7E-06 | 1 |
| transcription factor CP2-like 1 | TFCP2L1 | -4.11 | 1.1E-05 | 1 |
| UDP-galactose-4-epimerase | GALE | 2.83 | 1.1E-05 | 1 |
| lipase, member H | LIPH | 22.57 | 1.1E-05 | 1 |
| DEP domain containing MTOR-interacting protein | DEPTOR | -2.12 | 1.1E-05 | 0 |
| pleiomorphic adenoma gene 1 | PLAG1 | 6.15 | 1.1E-05 | 1 |
| slit homolog 1 (Drosophila) | SLIT1 | 6.54 | 1.2E-05 | 1 |
| UDP-N-acetyl-alpha-D-galactosamine:polypeptide N-acetylgalactosam | GALNT7 | 3.67 | 1.3E-05 | 1 |
| LIM domain only 3 (rhombotin-like 2) | LMO3 | 3.11 | 1.3E-05 | 1 |
| primary ciliary dyskinesia protein 1 | PCDP1 | -2.40 | 1.6E-05 | 1 |
| sodium channel, voltage-gated, type IV, alpha subunit | SCN4A | 2.54 | 1.7E-05 | 1 |
| dual-specificity tyrosine-(Y)-phosphorylation regulated kinase 4 | DYRK4 | -2.11 | 1.8E-05 | 0 |
| patched domain containing 4 | PTCHD4 | 4.40 | 1.8E-05 | 1 |
| TEA domain family member 4 | TEAD4 | -2.23 | 1.9E-05 | 0 |
| SMAD family member 9 | SMAD9 | -2.85 | 1.9E-05 | 1 |
| protocadherin 7 | PCDH7 | 4.11 | 2.2E-05 | 1 |
| SOGA family member 3 | SOGA3 | 3.15 | 2.9E-05 | 1 |
| spermatogenesis associated 18 | SPATA18 | 3.40 | 3.3E-05 | 1 |
| glutaminyl-peptide cyclotransferase | QPCT | 4.47 | 3.5E-05 | 1 |
| nucleoporin 62kDa C-terminal like | NUP62CL | -2.01 | 3.6E-05 | 0 |
| sarcoglycan, beta (43kDa dystrophin-associated glycoprotein) | SGCB | 2.35 | 3.7E-05 | 0 |
| sciellin | SCEL | 5.47 | 4.1E-05 | 1 |
| integrin, alpha 2 (CD49B, alpha 2 subunit of VLA-2 receptor | ITGA2 | 2.86 | 4.6E-05 | 1 |
| immunoglobulin superfamily, member 1 | IGSF1 | 7.30 | 4.7E-05 | 1 |
| kyphoscoliosis peptidase | KY | 2.25 | 4.7E-05 | 0 |
| RNA binding protein with multiple splicing 2 | RBPMS2 | -2.50 | 4.8E-05 | 1 |
| keratin 222 | KRT222 | 2.75 | 5.0E-05 | 0 |
| acid phosphatase, prostate | ACPP | 2.36 | 5.1E-05 | 1 |
| wingless-type MMTV integration site family, member 5A | WNT5A | 2.06 | 5.5E-05 | 0 |
| IKAROS family zinc finger 2 (Helios) | IKZF2 | 2.43 | 6.3E-05 | 0 |
| insulin-like growth factor binding protein 6 | IGFBP6 | 3.39 | 6.4E-05 | 0 |
| shroom family member 4 | SHROOM4 | 2.86 | 6.5E-05 | 0 |
| calpain 3 (p93) | CAPN3 | 2.36 | 7.0E-05 | 1 |
| serine-rich and transmembrane domain containing 1 | SERTM1 | -5.48 | 7.5E-05 | 1 |
| retrotransposon gag domain containing 4 | RGAG4 | 3.59 | 8.0E-05 | 1 |
| solute carrier family 26, member 8 | SLC26A8 | 2.95 | 8.7E-05 | 1 |
| Rho GTPase activating protein 36 | ARHGAP36 | 11.00 | 9.0E-05 | 0 |
| cytoplasmic polyadenylation element binding protein 3 | CPEB3 | -2.00 | 1.1E-04 | 0 |
| SH3 domain containing ring finger 2 | SH3RF2 | -2.15 | 1.2E-04 | 0 |
| GRAM domain containing 2 | GRAMD2 | -2.83 | 1.2E-04 | 0 |
| zinc finger protein 521 | ZNF521 | 2.53 | 1.4E-04 | 1 |
| receptor tyrosine kinase-like orphan receptor 2 | ROR2 | -3.15 | 1.4E-04 | 0 |
| pleckstrin and Sec7 domain containing 3 | PSD3 | 3.30 | 1.4E-04 | 1 |
| family with sequence similarity 70, member A | FAM70A | 2.95 | 1.4E-04 | 1 |
| synaptotagmin-like 5 | SYTL5 | 4.43 | 1.5E-04 | 0 |
| cadherin 3, type 1, P-cadherin (placental) | CDH3 | 4.27 | 1.5E-04 | 0 |
| pleckstrin homology-like domain, family A, member 1 | PHLDA1 | 2.42 | 1.5E-04 | 0 |
| AHNAK nucleoprotein 2 | AHNAK2 | 2.56 | 1.5E-04 | 0 |
| synaptic vesicle glycoprotein 2A | SV2A | 3.16 | 1.5E-04 | 0 |
| zinc finger protein 682 | ZNF682 | 2.29 | 1.7E-04 | 0 |
| neurobeachin | NBEA | 2.88 | 1.9E-04 | 0 |
| low density lipoprotein receptor-related protein 1B | LRP1B | -4.64 | 1.9E-04 | 0 |
| brain-specific angiogenesis inhibitor 3 | BAI3 | 2.11 | 1.9E-04 | 0 |
| adhesion molecule with Ig-like domain 2 | AMIGO2 | 2.26 | 2.0E-04 | 1 |
| SH3-domain GRB2-like (endophilin) interacting protein 1 | SGIP1 | 3.20 | 2.0E-04 | 0 |
| RAP1 GTPase activating protein | RAP1GAP | -3.27 | 2.0E-04 | 0 |
| ATP-binding cassette, sub-family A (ABC1), member 8 | ABCA8 | 3.93 | 2.1E-04 | 0 |
| family with sequence similarity 171, member B | FAM171B | 4.17 | 2.1E-04 | 0 |
| chromosome 1 open reading frame | C1orf130 | 2.01 | 2.2E-04 | 0 |
| uncharacterized LOC100129434 (ENSG00000233251) | LOC100129434 | -2.49 | 2.3E-04 | 1 |
| multiple C2 domains, transmembrane 2 | MCTP2 | 2.44 | 2.3E-04 | 0 |
| surfactant protein A1 | SFTPA1 | 4.33 | 2.3E-04 | 0 |
| histone deacetylase 9 | HDAC9 | 2.70 | 2.5E-04 | 0 |

^1^FC, fold change; ^2^p-value with FDR < 0.05 and fold change > 2;

^3^DEG, differentially expressed gene; 1, DEG according to Table 1; 0, not a DEG according to Table 1;

-, downregulated in FVPTCs *vs*. FAs.
